# Using longitudinal, multi-partner qualitative data to evaluate the implementation of a diabetes prevention and management intervention among South Asians Americans

**DOI:** 10.1186/s43058-025-00800-2

**Published:** 2025-10-30

**Authors:** Shahmir H. Ali, Deborah Onakomaiya, Nabeel I. Saif, Fardin Rahman, Farhan M. Mohsin, Sadia Mohaimin, Ashlin Rakhra, Shinu Mammen, Sarah Hussain, Jennifer Zanowiak, Sahnah Lim, Donna Shelley, Nadia S. Islam

**Affiliations:** 1 Department of Population Health, NYU Grossman School of Medicine, 180 Madison Ave, 8 Floor, New York, NY 10016 USA; 2 Saw Swee Hock School of Public Health, National University of Singapore, Singapore, Singapore; 3 School of Global Public Health, New York University, New York, NY USA; 4 SUNY Upstate College of Medicine, Syracuse, NY USA; 5 School of Osteopathic Medicine, University of the Incarnate Word, San Antonio, TX USA

## Abstract

**Background:**

Community-clinical linkage models (CCLM) display significant potential to address the unique, multi-level type 2 diabetes risk factors facing minoritized communities, such as South Asian Americans. However, there lacks a systematic, longitudinal evaluation of how such tailored CCLMs can be better implemented in dynamic, real-world settings. This study aims to leverage multi-partner insights, collected in real time, to explore the barriers and facilitators to implement a South Asian American diabetes management and prevention intervention (the DREAM intervention).

**Methods:**

The DREAM intervention, a two-arm randomized controlled trial, was implemented from 2019–2022; partners involved in its implementation were interviewed annually to understand their experiences of the program. Implementation partners included community health workers (CHWs), participating healthcare providers, community advisory board (CAB) partners, and research staff. The interview guide and subsequent deductive qualitative analysis was informed by the Consolidated Framework for Implementation Research (CFIR).

**Results:**

Overall, 78 interviews were conducted across four waves (2019–2022) with 5 research staff, 8 CHWs, 18 providers/clinic staff, and 12 CAB partners. CHWs adapted intervention characteristics by tailoring curriculum and implementation to patient needs, including personalized goal setting and shifting to remote delivery with COVID-19-related content. At the individual level, participants’ occupations, family dynamics, and technological capacity shaped engagement, while changing social, financial, and health contexts over time required CHWs to continually adjust support. Within the inner setting, partner roles and resource availability fluctuated, yet structured and consistent meetings facilitated communication and problem-solving. Outer setting influences, including shifting government and universities policies and the COVID-19 pandemic, required repeated adaptations, while CAB partnerships expanded community connections and services over time. Process-related findings underscored the evolving role of CHWs and research staff in planning and fidelity, with training shifting toward peer mentorship to build capacity.

**Conclusion:**

Findings revealed the pivotal role of programmatic adaptability and robust partner engagement in navigating dynamic contexts to support the diabetes needs of minoritized communities. The real-time, longitudinal approach taken for data collection and analysis was crucial in understanding how intervention changes were implemented and experienced, providing a model for similar implementation assessments.

**Supplementary Information:**

The online version contains supplementary material available at 10.1186/s43058-025-00800-2.

Contributions to the literature

- The study employed longitudinal qualitative data, involving repeated interviews with partners throughout the study, to uncover both persistent and changing programmatic barriers and facilitators over time.
- Findings emphasize that community-clinical linkage models designed for culturally minoritized communities must integrate a degree of structural flexibility and both build and sustain long-lasting partner relationships to support programmatic resiliency to resource changes.
- By documenting experiences and reactions to the intervention adaptations in real time, this study offers qualitative insights on partner responses that, in past studies, are at times missing or collected only at the end of implementation.

## Background

There is a pressing need to better evaluate implementation barriers and facilitators of multi-level health interventional strategies, particularly those aimed at addressing the complex determinants of non-communicable diseases such as type 2 diabetes (T2D). While the behavioral and health impact of such programs have been rigorously explored, systematically evaluating implementation processes and experiences shed crucial light on how such interventions can be disseminated and scaled up. Existing evidence-based strategies of T2D prevention and management, such as the National Diabetes Prevention Program’s (DPP) [1], often focus on promoting healthy lifestyle behaviors (namely diet, physical activity, and stress management), particularly through interventions in healthcare settings [2]. The reach and impact of DPP and other strategies are limited in minoritized communities (including racial and ethnic minorities) [3], in part due to challenges in ensuring adapted DPPs consider community-level variations in the socio-ecological risk factors for T2D [4] and are culturally appropriate (e.g. are responsive to and sensitive of the beliefs, customs, and other cultural characteristics of a community) [5].

However, community-engaged approaches have demonstrated potential for sustainable and impactful T2D prevention and management [6]. Community-clinical linkage models (CCLM), defined as “partnership models that connect health providers, community-based organizations, and public health agencies to foster patient access to preventive, chronic care, and social services,” have been tested as a strategy to advance cultural congruence in T2D prevention efforts [7]. A growing body of literature demonstrates that evidence-based interventions can be effectively disseminated to underserved communities when delivered through community health workers (CHWs), trained healthcare professionals that serve as a liaison between healthcare systems and patients, and often play a central role in CCLMs [8]. While not all CHW-led interventions are considered CCLMs (e.g., those that do not involve structured collaboration with healthcare systems or lack formal referral and feedback loops), CCLMs are typically characterized by intentional and structured partnerships across clinical and community sectors, often incorporating components such as social needs screening, referrals, and follow-up to address both medical and non-medical drivers of health. Indeed, CCLMs and CHW-led interventions have demonstrated effectiveness in diverse communities; specifically, integrating CHWs into team-based care can improve patient health-related behaviors (i.e., medication adherence, lifestyle changes) as well as address system-level barriers and social determinants of health [9, 10]. However, integrating CHWs into formal healthcare teams remains complex, with persistent challenges around recruitment, training, retention, and sustainable financing. CHWs are often employed outside of health systems, which can hinder coordination, limit role clarity, and reduce long-term integration [11]. Their effectiveness also depends on structured, ongoing training and close cultural and linguistic alignment with the communities they serve. As such, there is a crucial need to move beyond assessing impact alone and apply an implementation lens to better understand how CHW models can be feasibly scaled and sustained in diverse settings.

A majority of studies analyzed in a 2018 scoping review of 11 CHW-led community-clinical model interventions were focused on Hispanic and African American communities. South Asians, one of the largest Asian populations in the United States (US) and New York City (NYC), are disproportionately affected by chronic diseases, including T2D [12, 13]. A US study found a 27% prevalence of T2D among South Asians compared to 8% in non-Hispanic Whites [14], while a Canadian study reported South Asians were 3.4 times more likely than White individuals to develop the disease [15]. In cities such as NYC, with a particularly large community of Asian Indian, Bangladeshi and Pakistani Americans, the situation is further complicated by high rates of poverty, limited English proficiency, and poor access to culturally appropriate community resources [16, 17]. Thus, scalable, culturally tailored interventions that target diabetes prevention among South Asian Americans are warranted. The diversity within the South Asian community (by ethnicity, language, religion, and migration history) can influence healthcare engagement and the types of CHWs best suited to build trust; for example, among Muslim South Asian Americans (comprising a significant portion of the Pakistani and Bangladeshi community), gender concordance is especially crucial for effective CHW engagement [18]. To address this unmet need, the Diabetes, Research, Education, and Action for Minorities (DREAM) Initiative was implemented as a CHW-led community clinical linkage model focused on T2D prevention and management among South Asians living in NYC [19, 20].

Several factors have been shown to influence the successful implementation of CCLMs. For example, studies have found that CHWs’ strong ties with the patient community and ongoing training to empower CHWs with the tools needed in their role influence implementation success [21, 22]. Furthermore, a standardized approach to the hiring, supervision, and workflow of CHWs has also been suggested to facilitate successful implementation and sustainability of CHW-integrated CCLMs [10]. However, while CCLMs have been evaluated in the past, much of this work has centered around health-related outcome evaluations (e.g., improvements in HbA1C for CCLMs aimed at diabetes prevention and management) [11]. There is a notable lack of research on barriers and facilitators to the *implementation* of CCLMs, particularly understanding how these factors change over time, which is crucial to understand the scalability of such models and how well they can be applied in real-world settings. Indeed, longitudinal implementation analyses using both quantitative and qualitative methods have shown that community-based interventions are often shaped by an ever-changing landscape of socio-ecological factors impacting both patients and staff [23–25], which necessitate iterative adaptations to an intervention. Indeed, without a longitudinal lens to map both changes in these factors and subsequent interventional adaptations, crucial insights are either missed or simplified.

Prior research has revealed that initial challenges in communication between CHWs and provider teams can cause confusion regarding the roles and value of CHWs [22]. Similarly, a lack of mutual respect between CHWs and facility staff can further complicate the effective integration of CHWs into clinical settings. Logistical limitations have also been identified, such as CHWs reporting high burden of data collection and data management issues between partners [21]. In addition to these challenges, prior implementation studies on CCLMs identified a broader set of barriers and facilitators, including variability in CHW integration across settings, the need for sustained inter-organizational alignment, and the importance of ongoing training, trust-building, and clearly defined roles to support CHW effectiveness [21–23]. However, existing implementation evaluations of CCLMs have largely focused on cross-sectional analyses of CHW, provider, and research staff dynamics; there is a need to expand such implementation research to include other partners relevant to CCLM development, implementation, and evaluation (such as community-based partners and organizations). Moreover, while efforts have been made to longitudinally evaluate the implementation of community-based health interventions [23–25], including in the South Asian community [26], there is a crucial need to explore the barriers and facilitators to the implementation of CCLMs through a *longitudinal* lens, particularly to understand how implementation contexts evolve, and to examine the consistency and variation in barriers and facilitators in response to changing needs, external conditions, and partner dynamics. Although impact assessments of CCLMs have revealed significant improvements in diverse health outcomes [11], little is known about how the relationships between partners involved in a CCLM evolve over time.

Indeed, understanding how changes in roles, responsibilities, and partnerships within a CCLM affect the success and feasibility of an intervention is crucial for developing strong and sustainable models. The aim of this study is therefore to longitudinally evaluate barriers and facilitators to the implementation of the DREAM intervention in real time between 2019 and 2022, the period during which the intervention was carried out. Using annual interviews with key implementation partners, the study examines how shifting contextual factors and partner dynamics shaped implementation processes. Particular attention is given to how roles, relationships, and engagement evolved over time, and how these shifts influenced both the consistency and variability of barriers and facilitators. Importantly, the COVID-19 pandemic occurred during the implementation of the DREAM intervention, creating major social and logistical disruptions that required a wide array of adaptations [27]. The experience of responding to these disruptions makes the DREAM intervention a particularly well-suited case study for longitudinal evaluation of implementation under major temporal shocks.

## Methods

### DREAM intervention structure

Details on the DREAM intervention protocol have been described elsewhere [19, 20]. Briefly, the intervention was led by a research team at the New York University (NYU) Grossman School of Medicine and designed as a two-arm randomized controlled trial (RCT) focused on preventing and managing diabetes among South Asians in NYC with prediabetes or diabetes. Conducted in partnership with 20 community-based primary care practices (PCPs) serving South Asian communities, the trial included two integrated components. The first component focused on strengthening the use of Electronic Health Record (EHR) systems to better identify South Asians at risk for diabetes and to establish workflows for timely, effective intervention. Specifically, the research team trained PCP physicians and staff on EHR use and supported the implementation of patient registries and alerts within the system.

The second component embedded a team of CHWs into each PCP site to provide health coaching and support to patients with prediabetes and uncontrolled T2D. CHWs were recruited from the same neighborhoods as participants, shared ethnic and cultural backgrounds, and often belonged to overlapping social networks, such as the same religious institutions, community groups, or sports clubs. They underwent training on diabetes and related complications, motivational interviewing, and human subjects research. Some also held degrees in health-related fields (e.g., data science, social work) and had prior experience in nonprofit or health advocacy roles. CHWs delivered one-on-one and group-based education using a culturally adapted version of the DPP curriculum tailored to South Asian dietary practices and lifestyle behaviors [19, 20]. The curriculum included five monthly 60–90-min group sessions covering diabetes knowledge and complications, nutrition, physical activity, and stress management, delivered in participants’ preferred languages (e.g., Bengali, Urdu, Punjabi). In addition, CHWs conducted in-person visits approximately every two weeks, supplemented by up to ten follow-up phone calls, to support goal-setting, monitor progress, and address individual needs. CHWs also addressed social service needs using a structured screening and referral process. While a digital referral system was available, they most often connected participants directly to culturally appropriate resources such as employment assistance, language classes, immigration support, and food access through trusted South Asian-serving community organizations. All referrals and follow-ups were documented for tracking. The intervention was delivered over a six-month period, with adaptations made in response to COVID-19, including the use of virtual delivery formats and mailed materials.

The implementation of the study was further guided by a community advisory board (CAB) that informed the development, implementation, and evaluation of the study and optimized its impact, relevance, and sustainability. This advisory board was composed of organizations specifically serving the NYC South Asian community, including social service organizations, senior and community centers, as well as recreational groups. Members of the research team met CAB partners on a quarterly basis, and consultations were used to tailor the CHW educational curriculum, as well as to plan for activities catered towards intervention participants (e.g., exercise workshops). Importantly, based on the services provided by CAB partner sites, participants were also often referred to these organizations for social services.

The implementation of the DREAM intervention involved 4 major partners: 1) Research staff from NYU, comprised of program coordinators, administrative team, and other staff involved in coordinating all study activities, 2) CHWs who were employed by NYU and administered diabetes educational sessions with patients, 3) Providers and clinic staff from participating PCPs, and 4) CAB partners who were engaged throughout the study period.

### Data collection and analysis: the Consolidated Framework for Implementation Research (CFIR)

**Fig. 1:**
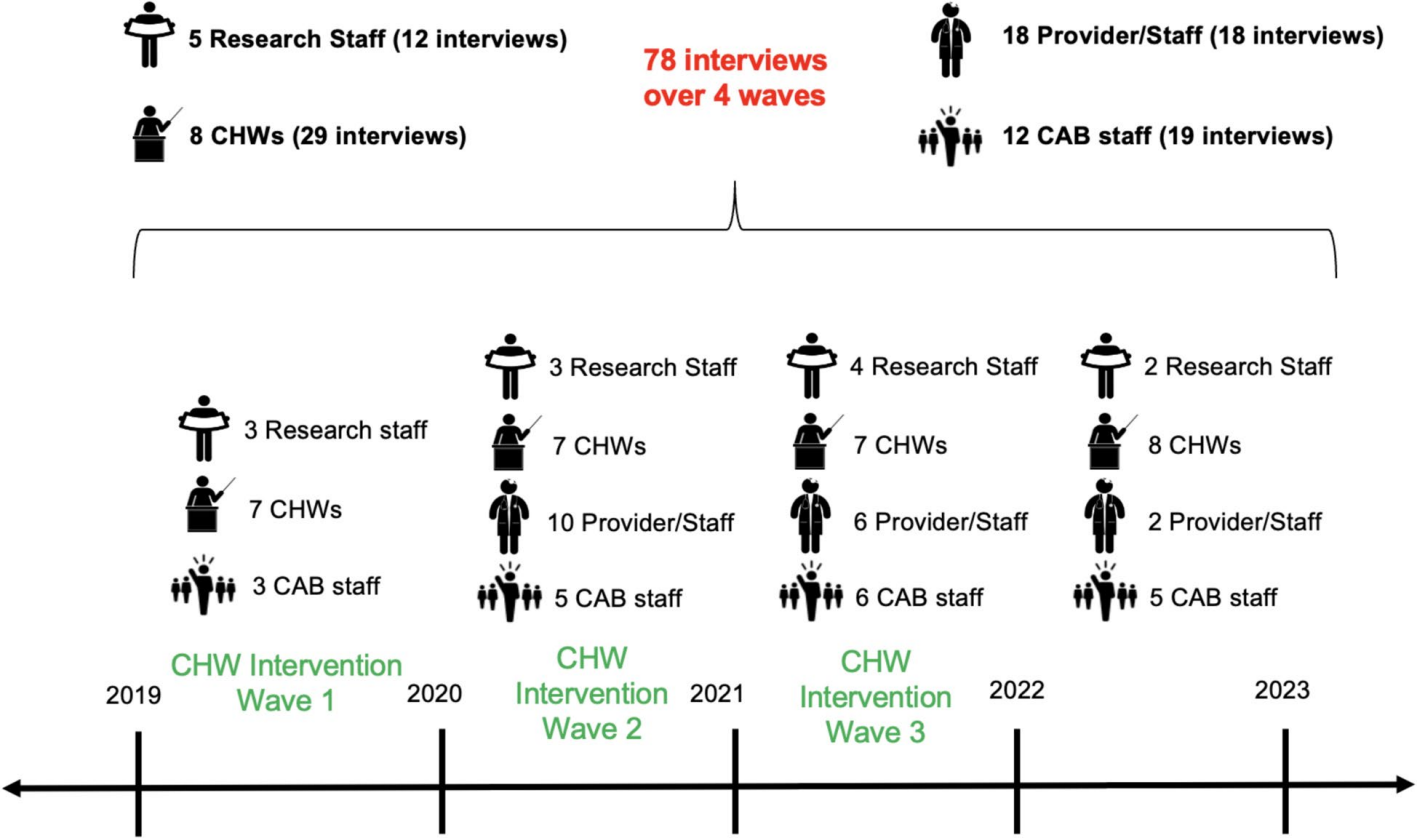
Timeline of DREAM implementation evaluation interviews

As part of the study, members from each implementation partner group were interviewed on an annual basis from the year of study commencement until study completion to understand their experiences with participating in the DREAM intervention (Fig. 1). To facilitate an open and comfortable environment for implementation staff to share their opinions, interviews were conducted by trained graduate-level students who were not members of the research team or partner sites implementing the study. All participating research staff and CHWs were invited and completed interviews. Additionally, at least one representative from each of the 7 participating CAB partners were annually invited for an interview. Staff turnover and changes in participating CAB partners contributed to yearly differences in the number of interviews conducted. At least one representative from each of the 14 participating PCPs (i.e. a provider, clinic staff, or both) was invited for one interview during the intervention wave in which they were involved. Ultimately, at least one staff member from all PCPs and CAB partners completed an interview. Annual invitations for clinic and CAB partners were sent via email and phone, with CHWs and research staff assisting in follow-up outreach as needed. Interviews could be conducted in-person or virtually via video-conference software.

The interview guide was developed from CFIR constructs [28]. This theoretically informed framework provides a tool for implementation scientists to deconstruct barriers and facilitators to the implementation of an intervention across five domains: intervention characteristics (the features and quality of the intervention, and its perceived complexity and adaptability), characteristics of individuals (the social circumstances and attitudes of the individuals in the intervention), inner setting (the setting and structure of the intervention, and the relationships between those implementing the intervention), outer setting (how the intervention’s relationship with the broader community and social context it is nested within), and process (e.g., meta-level considerations on how the intervention was planned, executed, and evaluated). For the DREAM intervention, CFIR domains were operationalized through tailored questions specific to each partner group. For example, CHWs were asked about adaptability in tailoring curriculum during Ramadan or for low-literacy participants, complexity in using online data collection software or shifting to virtual delivery, and outer setting influences such as pandemic-related social needs. Inner setting and process constructs were explored through questions on communication with providers, evolving CHW roles, and the shift from centralized to peer-led training models. This framework was chosen for data collection and analysis given its ability to comprehensively assess barriers and facilitators of an intervention in the context of essential pillars of implementation and further allow one to compare these barriers and facilitators with other implementation analyses of similar interventions.

Interviews were analyzed in real time (i.e., in the same wave of data collection as opposed to after completion of all waves of interviews across multiple years) to elucidate barriers and facilitators to the implementation process in the context of adaptations made throughout the intervention period; prior to the analysis, interviews were transcribed verbatim and analyzed using a codebook developed through CFIR. Specifically, we began with the five established CFIR domains and their sub-constructs, and then applied operationalized definitions tailored to the DREAM intervention (e.g., mapping relevant components, stakeholders, and processes under each subheading). These operationalizations were discussed among the analysis team and consensus was reached prior to beginning coding to ensure consistency and alignment with the intervention context. This initial codebook was then revised and expanded as it was applied to each transcript. The analysis primarily followed a deductive approach, with CFIR constructs providing the core coding structure, consistent with past implementation evaluations using similar deductive CFIR-driven coding [29, 30]. Data were systematically charted against these constructs, but flexibility was incorporated to allow sub-themes to be merged or stratified when interviews provided richer elaboration; for example, within the inner setting construct, communication themes were stratified by stakeholder group (staff-provider, staff-CHW, provider-CHW). This approach maintained the rigor of a structured framework while capturing important nuances in participant perspectives. Coding was conducted manually using Microsoft Word, which served as the primary platform for applying, organizing, and reviewing codes across transcripts.

To enhance reliability, each transcript was independently coded by two qualitatively trained graduate-level researchers; discrepancies in codes were deliberated to agree upon a final coding decision, and any remaining conflicts were resolved through the input of a third researcher. Interim analyses conducted at each wave of data collection were shared with the research team and used to inform ongoing intervention adaptations and guide subsequent activities. To obtain longitudinal insights from all coded transcripts, descriptive comparisons were made on how insights under each construct of the CFIR framework changed from findings of analyses conducted at different waves of data collection, including differences across partner group. To support this, codes and themes identified at each wave were reviewed at a meta-level to assess the consistency of themes over time and the emergence of new themes. Particular attention was given to identifying differences in themes both within and across stakeholder groups across waves. To provide additional context behind comments related to intervention adaptations, a number of major modifications made throughout the implementation period have also been reported using the FRAME framework [31], which systematically captures the timing, rationale, and nature of these changes.

Given the complexity of the qualitative data, we present the most salient findings for each CFIR construct and, where applicable, summarize overarching longitudinal insights within constructs. If notable subgroup-specific differences emerged over time, these are highlighted accordingly. To better display the key themes and stakeholder dynamics identified across the CFIR constructs, a visualization was developed following the full coding process. The directionality and strength of relationship dynamics were generated based on meta-level synthesis of the coded data, reflecting how frequently and closely stakeholders interacted across the intervention, as determined by the analysis team through iterative review and discussion.

## Results

**Fig. 2:**
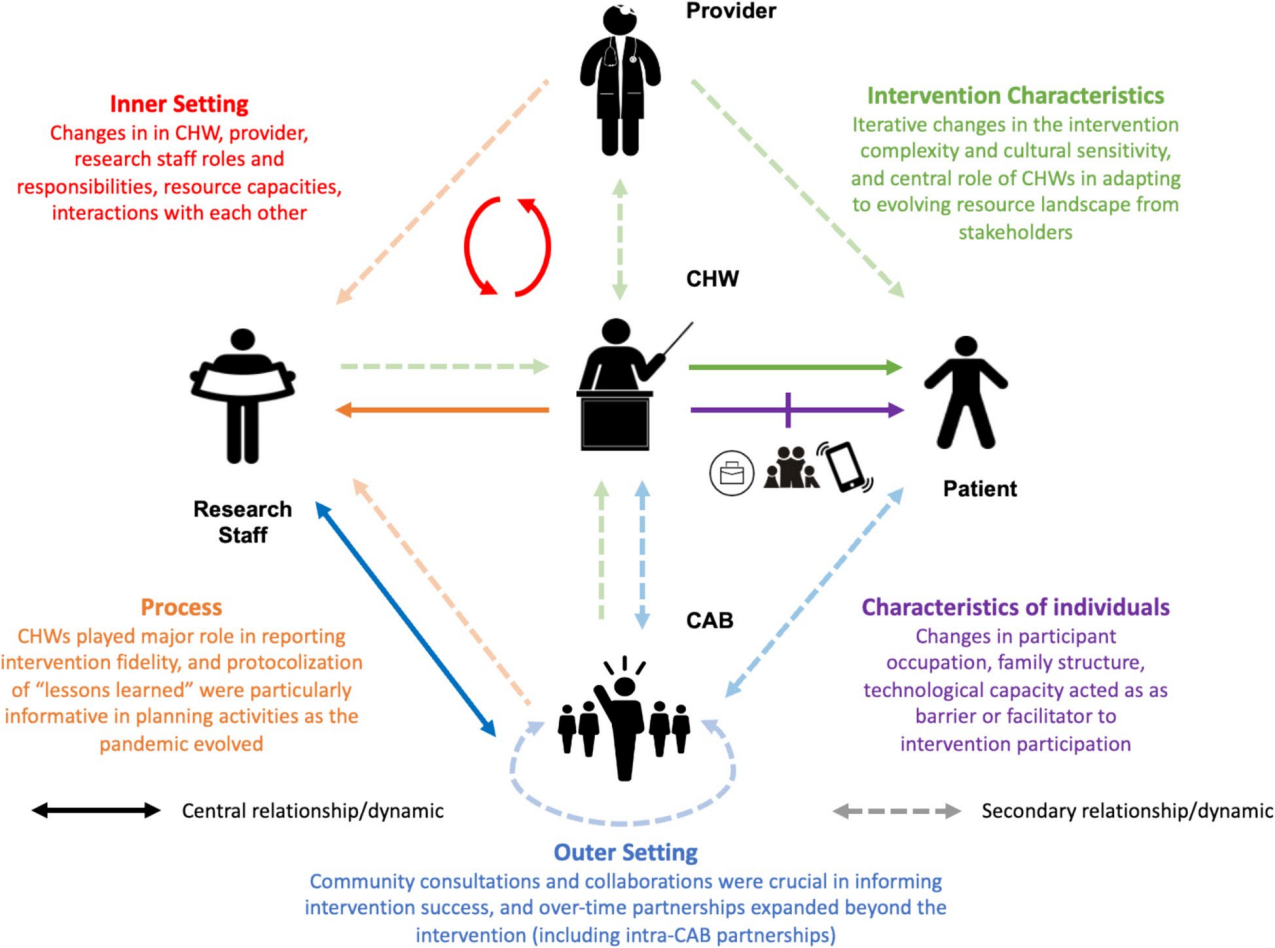
Visualization of key themes and relationships among DREAM intervention stakeholders under each CFIR construct

Overall, 78 interviews were conducted across 4 annual waves of data collection between 2019 and 2022. Interviews were conducted of 5 research staff (12 interviews), 8 CHWs (29 interviews), 18 providers or clinic staff across all 14 participating clinics (18 interviews), and 12 CAB organization staff across all 7 CAB partners (19 interviews). Most interviews were approximately 45 min, but ranged from approximately 20 min to 180 min during the duration of data collection. Figure 2 illustrates how key themes identified via the coding process (described in detail in this results section) intersected with overarching stakeholder dynamics. Arrows represent relationship dynamics identified through meta-level synthesis of coded data, solid arrows indicate primary (frequent, close) interactions, while dashed arrows reflect secondary relationships. Directionality shows the flow of engagement or information, indicating who was primarily involved or received insights related to each CFIR construct. Arrow colors reflect the CFIR domain most relevant to each interaction, linking stakeholder dynamics to specific implementation constructs. Additionally, key adaptations made to the intervention throughout the implementation period are summarized in Supplemental File 1, although additional details (particularly those related to adaptations during COVID-19) have been described elsewhere [27].

### Intervention characteristics

CHWs played a pivotal role in tailoring the DREAM intervention to the specific needs of their patients, drawing on their unique insights into barriers and facilitators encountered throughout the intervention to recommend and implement iterative changes. Their role included addressing curriculum and implementation issues to ensure the program remained relevant, appropriately engaging, and not overly complex. Key adaptations included providing personalized feedback on goal setting and modifying exercise tools for older patients with lower literacy levels. The most significant adaptation occurred when the intervention shifted to remote delivery during the COVID-19 pandemic. To support this transition, CHWs and staff developed resources such as videos, slides, and flyers that integrated COVID-19 information into the original diabetes-focused curriculum, as detailed in a separate analysis of the pandemic’s impact on DREAM implementation [27].

Longitudinal analyses revealed that perceived challenges to intervention delivery at the outset (particularly those related to cultural sensitivity) became less significant over time as CHWs, research staff, and other partners drew on successive years of implementation to make appropriate adaptations. For example, one research staff member reflected on implementing the intervention during Ramadan, a month of daily fasting and altered dietary practices observed annually by Muslims. Whereas Ramadan posed considerable logistical challenges in 2019, it was described as far less disruptive by 2020, reflecting how experience and accumulated adaptations reduced its impact on delivery.

#### Research staff A

*2019 Interview:* *“So Ramadan [is a] challenge from a programmatic standpoint—we have to alter the intervention during this one-month period based on what is comfortable for our participants as well as our CHWs who are fasting during this time, so we typically don’t hold sessions. Any meetings may be held on a 1 on 1 basis or as a home visit if both the CHW and the participant are willing to do so. And phone calls are all that we can do. And then the follow up for goal setting was to also be adapted for this period because participants are generally not able to exercise. But yeah, it does present some programmatic and logical challenges for CHWs– there’s really not much they can do.”*

*2020 Interview:* *“I think CHWs have developed a pretty good method for how to work with participants during Ramadan. They predominantly focus on nutrition and when you’re breaking fast. So healthy eating when you break fast and I think that’s kind of become the main thing that they talked about. Things slow down during that time, but I haven’t noticed it becoming a barrier, like it's just another reality that participants have figured out you know what’s the best way to do this intervention over the month and it's been working just fine.”*

### Characteristics of individuals

Analysis of the data revealed how participant characteristics (notably their occupation, family structure, and technological capacity) played a role in the implementation of the intervention. These personal attributes and needs regularly acted as facilitators or barriers to participation. For instance, several participants were taxi drivers or construction workers, for whom time availability was a frequent issue that made it difficult to schedule lessons. Family dynamics and structure could also act as either a barrier or a facilitator. For example, for many female participants, husbands often served as the main point of contact, so participation depended on coordination with husbands and the degree of material and emotional support they provided. Similarly, for older participants, adult children often managed all communication, which could either facilitate or hinder participation depending on family dynamics. Moreover, among participants with lower levels of education, partners reported noticing lower levels of digital literacy, which created challenges in coordinating session attendance or joining Zoom sessions (particularly after the transition to remote sessions) without assistance from a family member or CHW. This barrier was highlighted by both CHWs and participants, especially during the shift to virtual delivery due to COVID-19.

Longitudinal analyses revealed a changing social, financial, and health context which impacted the personal circumstances and priorities of participants (which included, among others, the COVID-19 pandemic, economic downturns, and changing federal/state policies related to health and immigration). Importantly, this ever-changing landscape of individual-level needs pushed CHWs and Research Staff to constantly reflect on how to keep the intervention both relevant and appropriate to an increasingly diversified range of participant level barriers and facilitators. For example, in reflecting on the most significant participant-level considerations in program implementation in 2019, 2020, and 2022, CHW A identified a wide range of priorities relevant to the biggest perceived challenges of each year:

#### CHW A

*2019 Interview: “[In the] area I work, the males are mostly construction people or taxi drivers—it’s really hard to bring them, because construction workers are working for seven days and they don’t have enough time for us. Secondly, the session schedule conflicts because they prefer something in the evening which is right after five o’clock or six o’clock. The problem is that we don’t have enough space—the doctor’s office gets crowded from four o’clock.”*

*2020 Interview: “We are having [a] hard time delivering sessions—I mean it was really hard, I [am] worried if I call someone—[they say] oh my god, we are dying, we don’t know if we will survive or die, [and] you are talking about your diabetes program? Forget about it! I was always ready for that and I told the whole team during our meeting. I said my approach was totally different. I didn’t even talk our program. I talked as a human being. Do you need any help? Just normal conversation. Once I feel that they are interested to talk, then I just [discuss] the program.”*

*2022 Interview: “We are not talking about mental health issues, which is really important. In the flood [referring to the COVID-19 pandemic], you’re not going to see the problem. Once the flood is gone, all the water is gone, then you will see the problem. We have to address these things very seriously for our community too. I know DREAM now is kind of gone, this is the last cohort. And we can come up with something – I ask my bosses at the office to seriously address mental health issues. It’s really important.”*

### Inner setting

Several factors within the inner setting influenced the implementation of DREAM, including partner roles, responsibilities, and available resources. Formal meetings between CHWs and research staff facilitated progress reporting and barrier resolution, enhancing support and goal feasibility. Communication between CHWs and providers was also crucial, with providers acting as intermediaries for recruitment and trust-building; in some cases, this even included participation in educational sessions. Providers found support from the research team valuable, especially early on (e.g., EHR tool training). Refresher trainings were later introduced in response to provider recommendations and were positively received. Initially, CHWs met individually with research staff, but many CHWs requested regular meetings with other CHWs as well. While CHWs felt their individual input was valued, they noted that group meetings allowed them to problem-solve among themselves before raising issues collectively with research staff. In response, a weekly CHW meeting was established and well received. Throughout the study, a major resource limitation raised by CHWs was time. Many devoted substantial effort to community outreach, which took time away from responsibilities such as education, data collection, and data entry. Both research staff and CHWs noted that limited technological proficiency initially made data entry challenging, though this improved over time. Student volunteers also provided critical support with data entry, alleviating burden on CHWs – a factor with implications for implementing similar interventions without such volunteer assistance.

Longitudinal analyses revealed that the roles and responsibilities of CHWs, research staff, and providers changed dramatically during the intervention. For CHWs and research staff, these changes were a result of being looped into new projects (in part to help cover salaries) or re-distribution of tasks when staff left or new staff joined. For providers and clinic staff, the onset of the COVID-19 pandemic resulted in a dramatic increase of workload, which led them to deprioritize their contributions to program implementation. Indeed, there was a large variability in the degree of interaction between providers and both CHWs and research staff. While the pandemic reflected a dramatic decrease in provider involvement with the program, later interviews (as the pandemic subsided) indicated re-engagement with providers. For example, CHW B reflects on the interactions with her assigned provider in 2020 and 2022:

#### CHW B

*2020 Interview: “I used to talk to her [provider] a lot but [after the] pandemic I am not going to the office—not very often we are talking to each other. Sometimes I called or left message for her saying this patient needs [something] or our participant needs letter of support, so this way [we are now] leaving messages to each other.”*

*2022 Interview: “I have very good communication with [the provider]—if I text, she will quickly respond. So, I would say, hello, can you check your patient’s medicine? So, this kind of way, I communicate.”*

### Outer setting

Over the course of the intervention, the outer setting remained dynamic, shaped by a diverse array of forces influencing implementation. Significant policy changes occurred at the local (including university), state, and national levels, many of which were driven by external pressures such as the COVID-19 pandemic. Meetings with CAB members provided a crucial lens into these external forces (particularly at the community and state levels) and helped research staff, and subsequently CHWs, to prepare and respond. In turn, CAB partnerships facilitated deeper embedding of the intervention within NYC South Asian communities by leveraging existing resources (e.g., hosting intervention activities in local senior centers or service organizations affiliated with CAB members) and by referring participants with health, social, or immigration-related needs to trusted community services.

Longitudinal analyses revealed that while CAB members often began their involvement in the intervention with some confusion of their roles and responsibilities, through successive years of engagement, meetings with CAB members helped foster partnerships and projects which expanded beyond the intervention. Importantly, these included partnerships among CAB members themselves, highlighting the wider impact of the DREAM intervention among partners in the health of NYC South Asian Americans beyond simple program related diabetes goals. For example, the 2020 and 2021 interviews of CAB member A describe the formation of new relationships with CAB member C:

#### CAB member A

*2020 Interview: “As a new CAB member, I'm not sure how this meeting thing works and how the resource sharing works… I know [CAB member B] very well—we always share resources and stuff like that, but with other organizations, I haven’t dealt with [CAB member C] before because they're in Coney Island. But I would be very interested to know if there are any programs… that we can help them with, or they can help us with.”*

*2021 Interview: “In the [CAB] meetings we can explore more about funding opportunities, collaborative [opportunities] like surveys, … [any] upcoming events that any specific organization is doing, and that helps us – let’s say like [CAB member C] is doing a food pantry and we have clients in Coney Island, we can see if [they] can support us a little bit, like ‘oh [CAB member C] would you like to stay for a couple of minutes after [the DREAM CAB meeting] and we can chat about this?’”.*

### Process

DREAM partners who played major roles in planning and maintaining fidelity of the intervention included CHWs and research staff. Early in the intervention, research staff reported that a major facilitator was providing CHWs with substantial support on curriculum implementation and protocols, particularly through multiple training sessions and opportunities to give feedback. In successive years, the nature of training and support evolved: in addition to receiving direct training from research staff, senior CHWs with more experience began helping to train newer CHWs. With regard to intervention fidelity, CHWs served as the primary lens through which research staff assessed whether the intervention was being implemented as intended, as they interacted directly not only with all other implementation partners (CAB members, research staff, and providers) but also with patients themselves. Feedback from CHWs in the field was therefore critical for monitoring fidelity, refining protocols, and reflecting on the broader impact of implementation, supplemented by insights from CAB members and providers.

Longitudinal analyses revealed that, while CHWs and (particularly) research staff emphasized the importance of and reliance on trainings, protocols, and existing resources to prepare for and implement the intervention in its early years, with years of successive protocol adaptations, there was greater emphasis on how the increasing flexibility of the program allowed for greater ease and success with implementation. For example, Research Staff B reflects on interventional protocols in 2019 and 2021:

### Research staff B

*2019 Interview:* *“We’ve also created a lot of protocols for our CHW to make it kind of easy to implement the program... protocols on recruitment, protocols on conducting the session, but also follow up and screening, so I feel like it’s a very well organized program.”*

*2021 Interview:* *“I think that implementing DREAM has, over the past year, gotten to be a little bit easier because we offer a lot of flexibility in the way that they are providing the health education sessions. CHWs' are no longer restricted by space, like available space at the provider practices [to conduct intervention sessions], and participants don't have to be at a certain place at a certain time, there's a lot of flexibility [now].”*

## Discussion

By longitudinally analyzing multiple perspectives on the implementation of the DREAM intervention, this study revealed the complexity and dynamic changes in the barriers and facilitators to a diabetes prevention and management program aimed at South Asian Americans. Findings from this evaluation also corroborated insights from past implementation evaluations of CCLMs, notably communication challenges and lack of consistent interactions between CHWs and providers (contributing to limited provider awareness of program goals) [22], as well as the significance (and subsequent workload) of CHWs in documenting implementation outcomes and providing feedback to inform adaptations of the program [21]. Across all five examined CFIR constructs, implementation partners highlighted each of their respective roles in ensuring the intervention could achieve and maintain its impact, efficiency, and relevance to the South Asian American community. Importantly, this involved balancing the need for iterative adaptations and strategic changes to the intervention structure and goals while maintaining a sense of consistency in the intervention structure to ensure reasonable fidelity to the intervention protocol. Indeed, by examining the resilience and adaptability of those involved, and the lessons learned, this study provides a crucial blueprint for scaling-up such an intervention into other settings or other communities with a similar health and social profile as South Asian Americans, and also ensuring interventions are prepared to handle dramatic social and resource shocks (such as those that occurred during the COVID-19 pandemic).

The use of longitudinal qualitative data, notably re-interviewing partners multiple times across the study, was an important novelty of the study in comparison to past qualitative applications of the CFIR framework [32] and implementation evaluations of CCLMs [11]. By comparing perspectives longitudinally, analyses were able to uncover the consistency of programmatic barriers and facilitators across time (e.g., patient-level obstacles related to occupation, family structure, and technological capacity) as well as crucial changes (e.g., greater/lesser interactions between partners, or changes in roles/responsibilities due to exigent circumstances, such as COVID or other community/organization-level forces). Likewise, by re-interviewing partners, the study was able to elucidate how iterative changes during the intervention were proposed, implemented, and perceived by partners. Importantly, unlike single-interview post-intervention qualitative evaluations (which often involve asking participants to reflect on multiple years of an intervention), this process allowed implementation stakeholders to reflect on much shorter, recent time frames, which helped to provide richer, nuanced qualitative data (particularly helpful for complex, multi-year interventions such as DREAM). As such, through the nuances extracted from this multi-year qualitative data, this study highlights the importance of considering multi-year perspectives in qualitative implementation evaluations of health interventions. Moreover, this study also contributes an important methodological case example of innovatively managing and synthesizing highly complex qualitative data (engaging diverse stakeholder groups involved in a multi-level CCLM across multiple years) to identify cross-cutting themes and generate salient longitudinal insights on implementation.

Moreover, there is a growing body of literature on how public health professionals can both better implement and evaluate adaptations made to interventions [33–35], which has been used to develop frameworks such as the Model for Adaptation Design and Impact (MADI) used in a previous analysis to evaluate adaptations made to the DREAM intervention during the COVID pandemic [27, 34]. However, unlike much of this past literature, which has focused on documenting the intervention adaptations themselves, the current study aimed to document the experiences of and reactions to the adaptations. Nonetheless, similar to the analyses of this study, past evaluations of intervention adaptations often also include sub-analyses focused on understanding the acceptability, fidelity, feasibility, and other multi-partner experiences of intervention adaptations [35]. Moreover, even if the intervention adaptations themselves have been longitudinally documented in real time, qualitative insights on how different partners responded to these adaptations are often cross-sectionally collected at the end of the study (if they are collected at all) [35, 36]. In this regard, the real-time longitudinal approach to data collection and analysis employed by this implementation evaluation may also benefit research aimed specifically at documenting intervention adaptations.

A notable finding (consistent across all years of data collection and partner perspectives) was the importance of flexibility in both the programmatic structure and goals of the intervention to maintain the intervention’s relevance to not only the changing landscape of factors contributing to South Asian diabetes prevention and management, but also the ever-changing social, health, and policy context in which the intervention was imbedded within. For example, due to changing resources and priorities, CAB members and providers were observed to straddle between the inner setting and outer setting of the intervention, in some years contributing to the program’s implementation, and in other years not being directly involved at all (instead reflecting on and shaping the wider social and health environment of participants). As a result, relationships between CABs and other stakeholders, such as CHWs and research staff, shifted over time; some strengthened through continued collaboration on community engagement efforts, while others became more peripheral during periods when CAB partners prioritized other organizational or community responsibilities. Similarly, the modality and format of the intervention (e.g., online vs in-person, frequency of interactions) as well as the curriculum content (e.g., integration of COVID-19 related content) also changed across the intervention, reflecting the most pressing needs and obstacles facing participants. Indeed, programmatic adaptability to community needs and long-term thinking (both reflected in the DREAM intervention) are core tenets of community-based participatory research (CBPR) [37], yet also reflect major challenges in research studies (which are often designed to target a pre-defined, funder-controlled set of outcomes, or may be inflexible to intervention changes out of evaluation and fidelity concerns) [38]. Findings emphasize that CCLMs designed for South Asian Americans and other similar communities must integrate a degree of structural flexibility, building and sustaining long-lasting partner relationships that can be resilient to programmatic and resource changes in order to optimize intervention implementation and impact. Likewise, funded research on such models should also allow for and embrace this variability.

While the key strengths of this study were its longitudinal structure and diversity in partners included in analysis, there are a number of limitations that must be acknowledged. First, due to staff changes, at times different representatives from each partner group were interviewed across the study period, whose insights about the program may have differed from other representatives from the organization. Second, due to scheduling and resource constraints with providers (which heightened substantially after the COVID-19 pandemic), providers were only able to be interviewed once throughout the duration of the study. Third, there was significant heterogeneity in size, structure, and level of engagement with the intervention across provider and CAB sites, which although had the benefit of providing diversity in perspectives for the implementation evaluation, also limited the ability to extract saturated themes from the data. Fourth, in 2022 an updated version of the CFIR framework was published [39]; since this was not published at the time the codebook was developed and applied, the original CFIR framework was used for analysis. Fifth, due to the limited sample size of the different intervention partner groups, survey-based quantitative implementation outcomes were unable to be collected and compared with the qualitative insights provided. Finally, the complexity of the longitudinal qualitative data (spanning multiple years, multiple stakeholder groups and a multi-level intervention) made it challenging to systematically track how every single construct examined evolved within and across groups over time, as participants sometimes reflected on multiple years within a single interview, themes varied in salience and meaning across roles and settings, and the timing and pace of change often differed between groups. As such, we present key longitudinal insights within each CFIR construct and prioritized a coherent, CFIR-guided reporting structure to highlight the most salient and cross-cutting changes across the intervention period.

## Conclusions

Overall, this implementation evaluation revealed specific, complex, multi-perspective insights on the barriers, facilitators, and changes to a diabetes prevention and management intervention tailored to South Asian Americans. For public health practitioners and policy makers, findings provide important considerations in how similar diabetes interventions tailored towards culturally and socio-economically diverse communities, such as South Asian Americans, should be developed, implemented, and evaluated, and how key partners (notably CHWs, research or administrative staff, providers and clinic staff, as well as community partners) can be involved. Importantly, the study offers a practical blueprint for scale-up to other settings and communities by highlighting the value of longitudinal engagement across diverse implementation partner groups, the utility of adaptive CHW training and support structures that reflect evolving staff capacities and relational dynamics, and the importance of delivering culturally grounded education that can flexibly respond to shifting participant needs and external disruptions. For researchers, findings pave the way for evaluating similar interventions (particularly post-pandemic interventions) in other communities facing parallel diabetes prevention and management related challenges to corroborate the applicability of the implementation related barriers and facilitators observed in this study.

## Supplementary Information

Supplementary Material 1.

## Data Availability

The data used and/or analyzed during the current study are available from the corresponding author on reasonable request.
