# Supplementary material for: Using longitudinal, multi-partner qualitative data to evaluate the implementation of a diabetes prevention and management intervention among South Asians Americans
Source: Implement Sci Commun. 2025 Oct 30;6:112. doi: 10.1186/s43058-025-00800-2 (PMC12574163; doi:10.1186/s43058-025-00800-2)
Supplement: Supplementary file 1 — Supplementary Material 1. [file 43058_2025_800_MOESM1_ESM.docx]

**Suppl. File 1: Summary of major adaptations made during implementation DREAM intervention, reported using FRAME**

| **Year** | **Modification planned?** | **Who made the decision?** | **Modification goal** | **What process was modified?** | **At what level of delivery?** | **Nature of modification** | **Reasons for adaptations** | **Baseline** | **Modification description** |
| --- | --- | --- | --- | --- | --- | --- | --- | --- | --- |
| 2019 | Unplanned | Research team | Improve provider engagement and communication | Format and frequency of provider-facing reports | Provider-facing | Adding new report components | Provider request for more detailed, timely patient updates | Simple enrollment data shared with limited intervals | New reports introduced with action plans to describe patient goals, progress toward goals, and types of social services requested |
| 2020 | Unplanned | Research team | Meet COVID-19 safety needs | Mode of delivery and curriculum content | Patient-facing (CHWs) | Substituting and tailoring | External factors (COVID-19) | In-person group sessions | Sessions shifted to remote; COVID-specific content expanded in curriculum |
| 2020 | Planned | CHWs and research team | Enhance cultural relevance and feasibility | Session scheduling and content | Patient-facing (CHWs) | Tailoring | Religious/cultural needs (Ramadan) | Standard DPP content and schedule | Curriculum and schedules adjusted for Ramadan; reduced frequency of interactions and evening sessions offered post-fast |
| 2021 | Unplanned | Research team and CHWs | Improve CHW support and efficiency | CHW check-ins and peer support | Staff-level (CHWs) | Adding elements | Implementation process need | Individual check-ins with research staff | Weekly CHW peer meetings implemented |
| 2021 | Unplanned | Senior CHWs and research staff | Build internal capacity and reduce trainer burden | CHW training process | Staff-level (CHWs) | Shifting roles | Resource reallocation (time/staffing) | Centralized research staff-led trainings | Fostering opportunities for more senior CHWs to train their newer peers |
| 2021 | Planned | CHWs and research staff | Address emergent participant needs | Curriculum content and referral services | Patient-facing (CHWs) | Adding elements | Emergent participant concerns (mental health, COVID-19) | Standard DPP curriculum with limited referral pathways | Content expanded to include mental health and COVID-19 support, including information on transmission, vaccination, and addressing mistrust. |
| 2022 | Unplanned | Research team | Reduce workload burden | Data entry and documentation | Staff-level (CHWs) | Adding elements | Resource constraints (time, staff capacity) | CHWs responsible for all documentation | Volunteers added to assist with data entry |
